# Frequency of Celiac Disease in Patients with Hypothyroidism

**DOI:** 10.1155/2012/201538

**Published:** 2012-03-28

**Authors:** Mojtaba Mehrdad, Fariborz Mansour-Ghanaei, Fereshteh Mohammadi, Farahnaz Joukar, Salimeh Dodangeh, Roya Mansour-Ghanaei

**Affiliations:** ^1^Department of Endocrinology, Guilan University of Medical Sciences, Rasht, Iran; ^2^Department of Medicine, Gastrointestinal and Liver Diseases Research Center (GLDRC), Razi Hospital, Guilan University of Medical Sciences, Sardarjangal Avenue, Rasht 41448-95655, Iran; ^3^Nursing and Midwifery, Gastrointestinal and Liver Diseases Research Center (GLDRC), Guilan University of Medical Sciences, Rasht, Iran; ^4^Gastrointestinal and Liver Diseases Research Center (GLDRC), Guilan University of Medical Sciences, Rasht, Iran

## Abstract

*Background*. Celiac disease (CD) is closely associated with other autoimmune endocrine disorders, particularly autoimmune thyroid disease. The aim of this study was to find the frequency of celiac disease in patients with hypothyroidism in Guilan province, north of Iran. *Methods*. A total of 454 consecutive patients with hypothyroidism underwent celiac serological tests antiGliadin antibodies (AGA), antitissue transglutaminase antibodies (IgA-tTG) and antiendomysial antibodies (EMA-IgA). Small intestinal biopsy was performed when any of celiac serological tests was positive. *Results*. Eleven (2.4%) patients were positive for celiac serology, and two patients with documented villous atrophy were diagnosed with classic CD (0.4%; 95%). Two patients with classic CD had Hashimoto's thyroiditis (HT) (0.6%; 95%). Six (54.5%) of 11 were suffering from overt hypothyroidism and 45.5% from subclinical hypothyroidism. Six (54.5%) had HT, and 45.5% had nonautoimmune hypothyroidism. *Conclusions*. In this study, prevalence of CD was lower than other studies. Most of the patients with CD were suffering from HT, but there was no significant statistical relation between CD and HT.

## 1. Background

Celiac disease (CD) is an immune-mediated enteropathy that develops in susceptible individuals upon ingestion of gluten-containing diet [1]. The classic definition of CD or gluten-sensitive enteropathy includes the following three features: villous atrophy; symptoms of malabsorption such as steatorrhea, weight loss, or other signs of nutrient or vitamin deficiency; resolution of the mucosal lesions and symptoms upon withdrawal of gluten-containing foods, usually within a few weeks to months [2]. The classic presentation of CD is a severe malabsorption syndrome with diarrhea, steatorrhea, and weight loss and possess antibodies against gliadin and especially tissue transglutaminase [2]. CD is closely associated with other autoimmune endocrine disorders, particularly autoimmune thyroid disease [1]. Among autoimmune disorders, increased prevalence of CD has been found in patients with autoimmune thyroid disease, type 1 diabetes mellitus, autoimmune liver diseases, and inflammatory bowel disease. Prevalence of CD was noted to be 1% to 19% in patients with type 1 diabetes mellitus, 2% to 5% in autoimmune thyroid disorders and 3% to 7% in primary biliary cirrhosis in prospective studies [3]. The pathogenesis of coexistent autoimmune thyroid disease and CD is not known [3]. The term autoimmune thyroid disease encompasses a number of different entities characterized by varying degrees of thyroid dysfunction and the presence of serum autoantibodies against thyroid tissue-specific components, such as thyroglobulin (Tg) and thyroid peroxidase (TPO) [4]. Hashimotos thyroiditis (HT) is defined by the presence of high serum thyroid antibody concentrations (TG and/or TPO) [5]. Likewise, several studies reported prevalence's ranging from 10% to 30% [6–9] for autoimmune thyroid disease, and from 4 to 19% for HT in patients with CD [7–10]. Screening patients with autoimmune thyroid disease for CD and vice versa can give an accurate perception to this association [1]. Early diagnosis and treatment lead to decrease of some complications as malabsorption, infertility, osteoporosis, and lymphoma in high-risk patients and improve drug absorption in these patients [5].

The purpose of this study is to determine the frequency of CD in the hypothyroid patients of Guilan province, north of Iran.

## 2. Patients and Methods

This descriptive study was done on 454 patients (49 men and 405 women, 10–85 years old, with mean age 39.46.13.54 yr) during February 2008 and February 2010. The patients were selected consecutively from endocrinology clinics of Rasht, the center of Guilan province. Serologic tests for celiac including EMA- IgA, IgA-tTG, and AGA-IgA were requested. Serum IgA-tTG (AESKULISA Tt.G-A (7503) Germany) and AGA-IgG (AESKULISA Glia-G Germany) were measured by enzyme-linked immunosorbent assay (ELISA), serum was diluted 1 : 100, and the results were expressed in Dutch unites per milliliter (DU/ML) [22]. Serum EMA (Euroimmun Germany) was determined by means of indirect immunofluorescence on frozen section of commercial slides of duodenum [5]. Seropositively was defined when one or more of measured antibody tests were positive, and all patients with at least one positive serologic test (EMA-IgA +, IgA-tTG >10 DU/ML and AGA-IgA >15 DU/ML  ) underwent upper endoscopy and at least four biopsies of second part of duodenum. The biopsy findings were classified by MARSH criteria (Table 1) [11]. 

HT was defined by the presence of thyroid antibodies and hypothyroidism (high TSH and anti-TPO+) [23]. Overt hypothyroidism was defined by fT4 = low, TSH > 10 MU/L, and subclinical hypothyroidism was defined by fT4 = NL, TSH = 5–10 MU*⁄*(L) [24].

**Figure 1:**
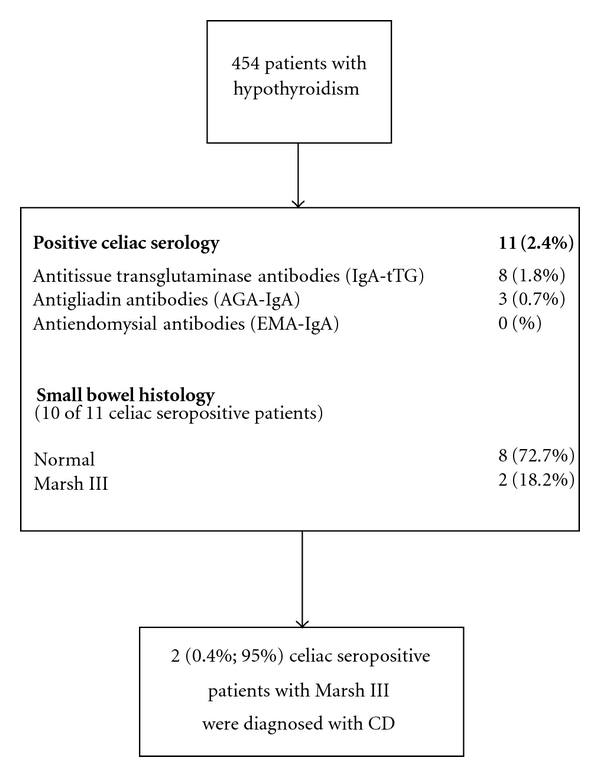
Flow diagram of patient recruitment and examination.

Small intestinal biopsies (at least 4 biopsies) were obtained during upper gastrointestinal endoscopy from second part of the duodenum with a spike forceps for histology. An experienced pathologist did the evaluation of all biopsied material according to the modified Marsh classification [25]. The study was approved by ethical committee of Gastrointestinal and Liver Disease Research Center (GLDRC) of Guilan University of Medical Science. Flow diagram of the study is seen in Figure 1.

### 2.1. Statistical Analysis

The data were analyzed by chi-square test. Continuous data having normal distribution are presented in means ± SD, and categorical data are presented in frequency rate and percentage. For all statistical analyses, a two-tailed *P* value <0.05 was considered significant.

## 3. Results

In this study, of 454 hypothyroid patients, 11 patients had positive serologic test for CD. Nine of them (81.8%) were female, and the other two were male (18.2%). Six (54.5%) of 11 were suffering from overt hypothyroidism and the rest 5 patients (45.5%) from subclinical hypothyroidism. Six (54.5%) had HT, and 5 (45.5%) had nonautoimmune hypothyroidism. Three had positive AGA-IgA and 8 had positive IgA-tTG. All of patients with positive serologic test underwent duodenal biopsy except for one patient, among these 10 patients, two patients (0.4%) relieved histologic change of celiac in small intestine (MARSH III), one with overt hypothyroidism and the other with subclinical hypothyroidism, both of them had classic CD. One patient with positive serologic test and iron deficiency anemia had normal biopsy and she classified as atypical CD. Seven patients with positive serologic test and normal biopsy had no gastrointestinal complains and were classified as having potential CD (Table 2).

## 4. Discussion

For many years, celiac sprue was defined by a set of classic standards for diagnosis. However, the combination of serologic, genetic, and histologic data has led to the identification of three other classes of CD.

Atypical or extraintestinal CD, where gastrointestinal signs/symptoms are minimal or absent and a number of other manifestations are present. Asymptomatic (silent) CD, where the small intestinal mucosa is damaged and CD autoimmunity can be detected by serology, but there are no symptoms. Latent, where individuals possess genetic compatibility with CD and may also show positive autoimmune serology that has a normal mucosa morphology and may or may not be symptomatic [26].

Two variants of what has been called latent CD have been identified: CD was present before, usually in childhood; the patient recovered completely with a gluten-free diet, remaining “silent” even when a normal diet was reintroduced. About 20 percent of such patients continue to have latent disease (asymptomatic with normal villous architecture) into adulthood, while the others redevelop variable degrees of villous atrophy [27]. Latency may be transient, and thus regular followup of such patients is warranted. A normal mucosa was diagnosed at an earlier occasion while ingesting a normal diet, but CD developed later [2].

Patients with potential CD have never had a biopsy consistent with CD but show immunologic abnormalities characteristic for the disease, such as a positive immunoglobulin A antibody to endomysium, tissue transglutaminase or increased intraepithelial lymphocytes (IELs) in the small intestine [28].

In this study, the total prevalence of CD including classic, atypical and potential in hypothyroid patients was 2.4% and prevalence of classic CD in hypothyroid patients was 0.4%. So the rate of prevalence in this study was lower than other studies [8]. Most of the patients with CD were suffering from HT, but there was no significant statistical relation between CD and HT; however, in Hadithi et al. study, there is relation between HT and CD [5], summarized prevalence of CD in hypothyroid patients in some studies (Table 3) [12–18]. Conversely, there is also an increased prevalence of immune-based disorders among patients with CD (Table 4) [5, 7–9, 19–21]. Female sex predominance was seen patients with celiac, but it was not significant (*P* = 0.626), however other reports show female sex predominance role in this disease [28]. Of 454 hypothyroid patients 338, (74.4%) had HT and the other 116 (25.6%) had nonautoimmune hypothyroidism. Both of the patients with CD had HT, one with subclinical hypothyroidism and the other with overt hypothyroidism. This finding supports the association between CD and autoimmune thyroid disease, but it was not a significant statistical relation (*P* = 0.408). All of the two patients showed gastrointestinal complains (one with flatus and the other with flatus and diarrhea). It is considerable that most of 11 patients with celiac including classic, atypical and potential had minor or no symptom and it can be concluded that the CD moves toward presenting minor gastrointestinal symptoms or asymptomatic. It is suggestive that the pattern of presentation of CD had altered over the past years [3]. At present, it is considerable that many patients with CD are asymptomatic [29]. The mean age of patients with CD was 43. 2.82 year; it was in agreement with previous reports [3]. Against other studies [5], in this study, patients with CD showed positive IgA-tTG and AGA more than positive EMA. However, Hadithi et al. found that most of patients with CD had positive EMA test [5]. The first published article about celiac disease in Iran is “High prevalence of CD in apparently healthy Iranian blood donors” in which 2000 healthy blood donors have been evaluated by EMA- IgA test; result of this study shows that prevalence of CD is 1 in 400 person [30]. In other study, prevalence of CD in Sari's adults has been evaluated in 2006; in this study, 1438 persons were tested for IgA-tTG and least prevalence of CD was determined 1 in 120 person [31]. Present study is the first study in Iran which determines frequency of CD in patients with hypothyroidism. The total prevalence of CD including classic, atypical and potential in hypothyroid patients was 2.4%, and prevalence of classic CD in hypothyroid patients was 0.4%. Most of the patients with positive serologic test for CD had HT (54.5%) and overt hypothyroidism (54.5%).

## 5. Conclusion

Screening high-risk patients for CD, such as those with autoimmune diseases, is a reasonable strategy given the increased prevalence. Treatment of CD with a gluten-free diet should reduce the recognized complications of this disease and provide benefits in both general health and perhaps life expectancy. CD and hypothyroidism can present with nonspecific symptoms. Thus, it is necessary to identify and treat a coexisting autoimmune disorder in order to adequately manage the primary disorder. Finally, the availability of serological screening tools and the possibility to prevent complications like osteoporosis or lymphoma in unrecognized patients with CD favor the screening of patients with HT for CD even in absence of symptoms. Screening of high-risk groups such as those with autoimmune thyroid disease is a reasonable strategy.

## Figures and Tables

**Table 1:** Modified marsh classification of CD [11].

Marsh type	Intraepithelial Lymphocytes per 100 Enterocytes	Crypts	Villi
0	<40	Normal	Normal
1	>40	Normal	Normal
2	>40	Increased	Normal
3a	>40	Increased	Mild atrophy
3b	>40	Increased	Marked atrophy
3c	>40	Increased	Absent

Type 0: normal mucosa; CD highly unlikely.

Type 1 (Infiltrative lesion): seen in patients on a gluten-free diet (suggesting minimal amounts of gliadin are being ingested); patients with DH; and family members of patients with CD. However, these patients need to be followed because they may convert to a type 3 lesion.

Type 2 (hyperplastic type): very rare; seen occasionally in DH.

Type 3 (destructive lesion): spectrum of changes seen in symptomatic CD.

**Table 2:** Clinical characteristics and symptoms in patients with positive serologic tests for CD.

Number	Age years	Sex	Type of hypothyroidism	Hashimoto thyroiditis	Clinical symptom	Positive test	Biopsy findings
1	45	Female	Overt	+	Flatus diarrhea	TGA	Marsh III
2	41	Female	Subclinical	+	Flatus	TGA	Marsh III
3	23	Female	Subclinical	+	IDA*	TGA	Normal
4	43	Female	Subclinical	+	−	TGA	Normal
5	56	Female	Subclinical	−	−	TGA	Normal
6	37	Female	Subclinical	−	−	AGA	Normal
7	21	Female	Overt	+	−	TGA	Normal
8	52	Female	Overt	−	−	TGA	Normal
9	61	Female	Overt	−	−	TGA	Normal
10	58	Male	Overt	+	−	AGA	Normal
11	12	Male	Subclinical	−	−	AGA	Not done

*Iron deficiency anemia.

**Table 3:** Prevalence of celiac disease (CD) in autoimmune thyroid disease.

Authors (year of publication)	Population screened	Prevalence of CD
Valentino et al. (1999) [12]	150 autoimmune thyroid disease	3.3%
Berti et al. (2000) [13]	172 autoimmune thyroid disease	3.5%
Volta et al. (2001) [14]	220 autoimmune thyroid disease	3.2%
Larizza et al. (2001) [15]	90 pediatric autoimmune thyroid disease	7.8%
Meloni et al. (2001) [16]	297 autoimmune thyroid disease	4.4%
Mainardi et al. (2002) [17]	100 autoimmune thyroid disease	2%
Ch'ng et al. (2005) [18]	115 Graves' disease	4.5%
Hadithi et al. (2007) [5]	104 Hashimoto's thyroiditis	4.8%

**Table 4:** Thyroid dysfunction in celiac disease (CD).

Authors (year of publication)	Population studied with CD	Prevalence of thyroid dysfunction
Collin et al. (1994) [19]	355	AITD* (19) 5.4%
		Autoimmune hypothyroidism (11) 3.1%
		Graves' disease (7) 2%

Counsell et al. (1994) [20]	107	Hyperthyroid (4) 3.7%
		Hypothyroid (11) 10.3%

Velluzzi et al. (1998) [7]	47	Hypothyroid (2) 4.3%
		Subclinical hypothyroid (3) 6.4%

Sategna-Guidetti et al. (2001) [9]	241	Hypothyroid (31) 12.9%
		Hyperthyroid (3) 1.2%
		Euthyroid AITD (39) 16.2%

Hakanen et al. (2001) [8]	79	AITD (11)13.9%
		Graves' disease (3) 3.8%
		Hypothyroid (8) 10.1%
		Subclinical thyroid disease (8) 10.1%

Carta et al. (2002) [21]	39	Subclinical hypothyroid (3) 7.7%

Hadithi et al. (2007) [5]	184	Euthyroid (10) 5%
		Overt hypothyroidism (22) 12%
		Subclinical hypothyroid (7) 3.8%

*Autoimmune thyroid disease.

## Abbreviations

fT4: Free thyroxine
TSH: Thyroid stimulating hormone
IELs: Intraepithelial lymphocytes
TPO-Abs: Antithyroid peroxidase antibodies
Tg: Thyroglobulin
IgA-tTG: Anti-tissue transglutaminase antibodies
HT: Hashimoto's thyroiditis
CD: Celiac disease
AGA-IgA: Anti-gliadin Antibodies
EMA-IgA: Anti-endomysial antibodies.
